# Evaluation of person-centred care after hip replacement-a controlled before and after study on the effects of fear of movement and self-efficacy compared to standard care

**DOI:** 10.1186/s12912-016-0173-3

**Published:** 2016-09-09

**Authors:** Lars-Eric Olsson, Elisabeth Hansson, Inger Ekman

**Affiliations:** 1 Institute of Health and Care Sciences, Sahlgrenska Academy, University of Gothenburg, Box 457, SE 405 30 Gothenburg, Sweden; 2 Gothenburg Centre for Person-Centred Care (GPCC), University of Gothenburg, Gothenburg, Sweden; 3 Department of Orthopaedics, Sahlgrenska University Hospital, Gothenburg, Sweden

**Keywords:** Person-centred care, Person-centered care, General self-efficacy, Tampa Scale of Kinesiophobia, ASA classification, Total hip replacement, Rehabilitation, Health plan, Planned surgery management, Nursing care

## Abstract

**Background:**

The goal of total hip arthroplasty (THA) is optimal pain relief and a normalized health-related quality of life. Anxious patients describe more pain and more difficulties than non-anxious patients during rehabilitation after THA. The aims of the present study were twofold: (1) to identify vulnerable patients using the general self-efficacy scale (GSES) and the Tampa scale for Kinesiophobia (TSK), and (2) to evaluate if person-centred care including the responses of the instruments made rehabilitation more effective in terms of shortening hospital length of stay.

**Methods:**

The design of the study was quasi-experimental. Patients scheduled for THA, a control group (*n* = 138) and an intervention group (*n* = 128) were consecutively recruited. The intervention was the provision of person-centred care which was designed to reduce the negative effects of low self-efficacy and high levels of pain-related fear of movement.

**Results:**

Patients with low GSES in the intervention group had shorter length of stay (LoS) by 1.6 days (95 % CI 0.16–3.15) *p*-value 0.03. Patients with high TSK in the intervention group had shorter LoS by 2.43 days (95 % CI 0.76–4.12) *p*-value 0.005. For patients who had both, the reduction of LoS was 2.15 days (95 % CI 0.24–4.04) *p*-value 0.028.

**Conclusions:**

The GSES and the TSK instrument were found useful as tools to provide information to support patients which reduced the LoS by 1.67 days in the whole intervention group (95 % CI 0.72–2.62) *p*-value 0.001. More importantly, vulnerable patients such as ASA group 3 probably gained the most from the extra support, they had a reduction with 6.78 days (95 % CI 2.94–10.62) *p*-value 0.001.

## Background

### Person-centred care

Today, person-centred care is widely advocated as an important component of care in varied contexts [1, 2]. A key element in person-centred care is the dialogue between the health care professional and the patient: a dialogue with the patient that emphasizes shared decision-making as opposed to talking to (or informing) the patient [3]. The aim of this dialogue is to come to a mutual understanding and agreement of the planned care while medical decisions as always remain the physician s responsibility. This is the first step of person-centred care and the foundation for the *partnership* that commonly results in an individualized health plan formulated by the health care professional and the patient together (relatives are often involved as well). The health plan includes both short-and long-term goals for the patient along with the actions needed to reach each goal. The plan is a “living” document specific to each patient, in which the goals and actions are tracked and revised over time. In earlier controlled studies the implementation of person-centred care shortened hospital length of stay (LoS) by 30–50 %, significantly reduced uncertainty concerning illness and treatment, and decreased the number of medical complications observed [4–7]. In addition, a recent randomised controlled trial demonstrated significant increased self-efficacy after implementation of person-centred care in primary care [8]. Consequently the present discussion is not whether person-centred care should be implemented in hospital wards and primary care settings or not, but rather to refine and make it even more useful and effective.

### Self-efficacy

Self-efficacy can be described as confidence in one’s ability to cope [9, 10]. Effective patient self-management needs to address patients’ confidence in their ability to perform specific activities rather than just convincing them of the value of such activities [11]. The correlation between self-efficacy and rehabilitation outcomes in patients with chronic pain was found to be an important component of therapy [12]. A relationship between self-efficacy and pain self-management and coping strategies was also found in community residents with chronic pain [13]. Self-efficacy was shown to be more important than pain intensity and duration in determining disability among patients with chronic musculoskeletal pain [14, 15].

Bandura claims that efficacy expectations determine how much effort a person will expend and how long he or she will remain committed when resistance is encountered (Bandura 1997). Thus, self-efficacy is an important aspect in rehabilitation when patients are put in unfamiliar situations that challenge their ability to care for themselves.

### Fear of movement

Pain is a salient feature and one of the main factors leading to variable outcomes after surgery [16]. The available literature on the topic provides strong evidence of the influence of catastrophizing on emotional, functional, and physiological responses to pain [17]. During the first 1 or 2 days after Total hip arthroplasty (THA) surgery, the postoperative pain intensity can be described as “the worst imaginable” and may be associated with fear and a feeling of faintness [18–20]. Such pain can invoke a fear of movement, which has been shown to partially mediate the effects of pain intensity on disability at the onset of lower back pain [21]. Fear and avoidance of activity play a role in fostering disability in whiplash-associated disorders, and fear reduction produces a significant effect on outcomes [22]. A more complete understanding of patients’ experience of pain gained through validated measuring tools could lead to the optimization of patient care [16].

Patients can respond to fear of movement in one of two ways: they can choose to confront the situation or to avoid it. Confrontation leads to reduced fear, while avoidance will increase the fear. Pain-induced avoidance behaviour has two components: the avoidance of pain itself and the avoidance of painful activities [17]. Those who confront situations associated with pain experience less frequent pain, a shorter duration of pain, less fear of pain, and less fear of injury than those who avoid the same situations [23, 24]. Accordingly, the TSK may be a tool for preoperative assessment because it provides information about the levels of support and pain relief needed.

### Total hip arthroplasty (THA)

The goal of THA is optimal pain relief and an essentially normalized health-related quality of life. In most cases, the need for THA is related to osteoarthritis of the hip. When pain or functional limitations become moderate to severe, THA is indicated.

The current orthopaedic procedures are extremely effective, but patient rehabilitation to functional daily life can be challenging for health care professionals. Reportedly, anxious patients describe more pain and are less satisfied with pain relief compared to patients who are not anxious; therefore, anxious patients may experience more difficulties during rehabilitation after THA [25, 26]. Anxiety has also been found to correlate with a lower health-related quality of life [27]. Thus, improving self-efficacy and reducing fear of movement among patients who undergo THA are important goals for health care professionals.

We hypothesized that low self-efficacy and high levels of pain-related fear of movement have a negative effect on rehabilitation after THA. We also hypothesized that these negative effects could be reduced by person-centred care. The aims of the present study were twofold: (1) to identify vulnerable patients using the general self-efficacy scale and the Tampa scale for Kinesiophobia and (2) to evaluate if person-centred care including the responses of the instruments made rehabilitation more effective in terms of shortening hospital length of stay after THA.

## Methods

**Fig. 1:**
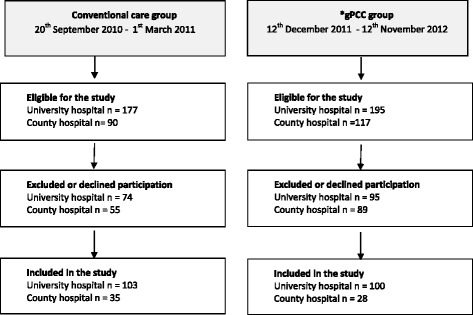
Study flow chart. *gPCC: Gothenburg person-centred care

Between September 2010 and November 2012, 266 patients admitted for planned primary THA were enrolled in the study from two designated Swedish orthopaedic departments, one in a county hospital and one in a university hospital. Detailed methods have been described elsewhere [28]. Briefly, the study had a quasi-experimental, controlled, before-and-after design. A control group (*n* = 138) were consecutively recruited between the 20^th^ September 2010–1^st^ March 2011 and an intervention group (*n* = 128)) were consecutively recruited between the 12^th^ December 2011–12^th^ November 2012. Patients' were included if they were scheduled for THA, able to complete the questionnaires, and willing to participate (Fig. 1).

### Standard care

During the examination at the out-patient clinic, all patients were examined by a physician and a RN. The patients answered questionnaires about their living circumstances, physical abilities and filled out surveys such as the GSES, TSK. They received standardized information including peri-operative routines and postoperative training based on hip replacement patients in general. Thus, they were all told that the expected LoS would be 4–5 days not considering the individual patients resources. All oral information was the same as in the written booklet which all patients got in order to have the possibility to repeat the information.

### The intervention

The patients in the intervention group did not receive standardized information, instead, all information was based on their own prerequisites. Evidence-based guidelines, clinical knowledge and patients’ individual prerequisites were combined with forming a partnership with professionals. The first step in establishing the partnership was for a RN specialized in surgical care to obtain a narrative from each patient, covering the patient’s everyday life, resources, motivation, and goals; patients were also asked to fill out the General Self-efficacy (GSES) and Tampa scale of kinesiophobia (TSK) questionnaires. Next, the RN made a tentative, detailed gPCC health plan based on the narrative, the medical examination, and the self-reported results of the GSES and TSK surveys. The gPCC health plan specified each patient’s short-and long-term goals, resources, special needs, and plan for recovery after discharge. It also included a predicted hospital LoS based on individualised data. The tentative health care plan was included in the letter provided to the patient at the outpatient clinic appointment 2 weeks before surgery. The health plan was discussed with the patient and finalized when an agreement was reached between the professionals and the patient.

When the patient was admitted to the ward the day before surgery, the RN at the ward confirmed with the patient that the plan still was valid. The nurses and physiotherapists were encouraged to be aware of each patient’s GSES and TSK from the beginning and to act on it. The patients were helped to familiarise themselves in the situation and to achieve their personal goal by emphasising their personal resources and capabilities documented in the health plan. We hypothesized that if the patients were aware of that the nurses new of their personal concerns, their confidence would be strengthened. Pain and fear interact in a negative way and usually we only target pain although targeting both probably is more effective. The nurses facilitated for the patients to describe their concerns and discussed this with them. Some patients based their concerns on troublesome stories they heard, or had misunderstood something while others just felt an anxiety and fear in general. For patients with high fear of motion they were encouraged to talk about their reason for fearing movement. Some patients for example believed the prosthesis needed to settle before it could be treaded on while others interpreted the pain as a sign that something was wrong. Some patients thought the training was rushed so they could be discharged earlier. For all patients in the intervention group it was very important that a rapid recovery was the safest way and would give the best results for them.

### Instruments

#### General self-efficacy scale (GSES)

The GSES scale consists of 10 items rated on a four-point Likert scale as follows: 1 = not at all true, 2 = hardly true, 3 = moderately true, 4 = exactly true. In this study, we used the Swedish version of the GSES [29]. The cut-off for low self-efficacy in the present study was set at ≤ 30 points based on the mean value from the database available at the GSES home page, which includes 19,896 values with a mean of 29.6 points [30]. In case of missing responses, we replaced up to two missing responses with the median value at the individual level [30].

#### Tampa Scale for Kinesiophobia (TSK)

The TSK is one of the most frequently used measures for fear of movement [23]. We used the validated Swedish version of the TSK instrument in this study [31]. The TSK scale consists of 17 items rated on a four-point Likert scale with scoring alternatives ranging from’strongly disagree’ to’strongly agree.’ Values for the 17 sub-questions were summed to yield a total value, which could range between 17 and 68. The values for questions 4, 8, 12, and 16 were reversed before summation. The measured margin of error has been estimated as 3 points: that is, a value between 34 and 40 (37 ± 3) [32]. In the present study, for high fear of movement the cut off was set at ≥ 40; we replaced up to two missing responses with the median value at the individual level.

#### American Society of Anesthesiologists” classification system (ASA)

The ASA classification system, which comprises six categories, is used worldwide for assessing the medical fitness of patients before surgery [33]. Patients scheduled for planned surgery commonly belong to one of three categories: (1) healthy, (2) mild systemic disease, or (3) severe systemic disease. The patients in this study were classified by the anaesthesiologist responsible for anaesthetizing patients during the surgical procedure.

### Outcomes

The primary endpoint of the study was the number of days spent in the hospital relative to the self-rated GSES and TSK scores. The hospital LoS was compared between the control group and the intervention group for patients scoring ≤ 29 on the GSES and/or ≥ 40 on the TSK. The relation between LoS and ASA category was also studied.

### Statistics

An audit of hospital records showed the combined mean hospital LoS from the two departments for the year 2009 was 7.4 days (SD 5.01). We decided to use the mean LoS from the control group (7.01) for our power analysis, and we estimated that 99 patients would be required in each group to achieve 80 % power to detect a 2-day reduction in LoS at a significance level of *p* < 0.05. Variables between-groups were analysed using Students *T*-test with 95 % confidence intervals, Fisher’s exact test for dichotomous variables, the Mantel–Haenszel Chi-squared test for ordered categorical variables, the Chi-squared test for non-ordered categorical variables, and the Mann–Whitney *U*-test for continuous variables. The data were analysed using SPSS version 19.0 for Windows (SPSS Inc, Chicago, IL, USA).

## Results

A total of 266 patients were enrolled in this study between September 2010 and November 2012. The control and intervention groups consisted of 138 and 128 patients, respectively. Baseline characteristics of the study cohort are shown in Table 1.

**Table 1:** Patient baseline data collected before surgery

Data	Control *n* = 138	gPCC *n* = 128	*P*
Female/male	89/49	83/45	0.9
Mean age	66	68	0.1
Standard Deviation	13,9	12	
Living			
with someone	90	68	0.6
alone	46	56	
Employment status			
Employed	32	33	0.2
Retired	84	79	
Disability pension	16	5	
Other	3	6	
Contact with relatives			
Weekly	129	120	0.8
Weekly to monthly	6	4	
< monthly	2	2	
Home nursing			
Yes	1	4	0.1
No	132	120	
Emergency medical alarm at home			
Yes	15	9	0.7
No	97	113	
Number of co-morbidities			
Median	1	1	0.06
Min	0	0	
Max	6	9	
ASA grade			
1	36	22	0.1
2	75	69	
3	27	16	
Using naturopathic preparation			
Yes	116	107	1.0
No	19	17	
Type of living			
Flat	62	74	0.02*
House	75	52	
Service flat	1	2	
Need of assistance from relative			
Yes	71	67	1.0
No	57	54	
Need of community home help			
None	120	115	0.7
Once a week	6	6	
Daily or more	7	4	
Assistive aids for personal use such as pincers, seat cushions and so on			
Yes	46	51	0.4
No	44	61	
Pre-fracture independence^b^			
80–100 %	132	117	0.9
60–79 %	3	7	
< 60 %	3	3	
Mean	92	92	
SD	13	16	
Type of walking aid			
None	44	42	0.5
Crutches	64	54	
Walking frame	14	19	
Wheel chair	3	4	
Previous hip replacement in contralateral hip			
Yes	47	32	0.4
No	91	95	
Feeling healthy			
Yes	78	77	0.7
No	19	16	

^a^Ceder scale

^b^Functional Recovery Scale [40]

*significant

The missing data for some of the variables were not regarded to impact the overall study results

*gPCC* Gothenburg person-centred care

The majority of the patients were women (172 women vs. 94 men); the mean age was 66 years in the control group and 68 years in the intervention group. There were no significant differences between the groups in terms of baseline characteristics except that more patients were living in flats in the intervention group. Almost all patients were self-sufficient before surgery despite the troubling hip measured by the functional recovery scale.

The occurrence of low GSES and high TSK in the control and intervention groups is shown in Table 2. Low GSES and high TSK scores were very common in both groups of patients scheduled for THA surgery around 1/3 had low GSES and about half had high fear of movement (Table 2).

**Table 2:** Occurrence of low GSES and high TSK in the entire study group of 266 patients

	Control group (*n* = 138)			Intervention group (*n* = 128)			
	*n*	Mean (SD)	Median (Min-Max)	*n*	Mean (SD)	Median (Min-Max)	*P*-value
GSES ≤ 29^a^	36 (26 %)	25.3 (4.2)	27 (11–29)	40 (37 %)	24.3 (5.1)	26 (10–29)	0.3
TSK ≥ 40^b^	70 (52 %)	46.9 (5.3)	46 (40–64)	63 (59 %)	43.2 (5.5)	45 (40–60)	0.4

^a^Some of the GSES questions were considered difficult; as a result, 19 patients in the control group and 10 in the intervention group left some or all of them blank and therefore had to be excluded

^b^ *Regarding the TSK*, *8 patients in the intervention group did not answer all questions and had to be excluded*

SD standard deviation, GSES general self-efficacy scale, TSK tampa scale of Kinesiophobia

The result was arranged as incremental distribution to make the result more visible. In the control group 30 % had low GSES vs. 34 % in the intervention group. Among patients who scored low on the GSES, there was a significant shift toward a shorter LoS in the intervention 1.6 days shorter than in the control group. The range was 6 days shorter in the intervention group compared to the controls (Table 3).

**Table 3:** Incremental distribution and overall LoS in patients with low GSES (≤ 29)

Control Group *N* = *119*								
	LoS ≤ 5 days			LoS 6–9 days				LoS ≥ 10 days
GSES ≤ 29	*n* = 36	15 (42 %)		14 (39 %)				7 (19 %)
Intervention group *N* = 118								
GSES ≤ 29	*n* = 40	23 (58 %)		14 (35 %)				3 (1 %)
Overall LoS in the groups with low GSES ≤ *29*								
		Mean LoS	SD	Range	Difference	95 % CI		*P*-value
Control Group	*n* = 36	7.31	3.9	18 days	1.60	0.16	3.15	0.03
gPCC Group	*n* = 40	5.70	2.3	12 days				

LoS length of stay, GSES general self-efficacy scale, gPCC Gothenburg person-centred care, SD standard deviation

Surprisingly many patients had a high TSK, around 50 % in both groups. Among patients who scored high on the TSK, there was a significant shift toward a shorter LoS in the intervention group compared with the control group. In the sub-group of patients with high TSK the LoS was shifted one step from increment LoS ≥ 10 days to increment LoS 6–9 days. However, the difference in LoS between the groups was large (2.43 days) and the variation among the controls showed by SD and range was a big. Patients in the control group who had high TSK, had the longest LoS and the highest mean seen in any of the sub-groups, almost 8 days.

There was a correlation between TSK and LoS indicating that high fear of movement is a predictor for LoS which can be influenced (Table 4).

**Table 4:** Incremental distribution and overall LoS in patients with high TSK (≥ 40)

Control Group *N* = 138								
	LoS ≤ 5 days			LoS 6–9 days				LoS ≥10 days
TSK ≥ 40	*n* = 70	35 (50 %)		21 (30 %)				14 (20 %)
Intervention group *N* =120								
TSK ≥ 40	*n* = 63	36 (57 %)		25 (40 %)				2 (3 %)
Overall LoS in the groups with high TSK ≥ 40								
		Mean	SD	Range	Difference	95 % CI		*P*-value
Control Group	*n* = 70	7.91	6.4	41 days	2.43	0.76	4.12	0.005
gPCC Group	*n* = 63	5.48	2.2	12 days				

LoS length of stay, TSK tampa scale of Kinesiophobia, gPCC, Gothenburg person-centred care, SD standard deviation

In total 20 % of all patients scored low on GSES and high on TSK, 17 % in the control group vs.21 % in the intervention group. The number of days was reduced by more than 2 days in the intervention group relative to the control group (*p* = 0.028). There were almost twice as many patients in the increment LoS ≤ 5 days in the intervention group (60 %) compared to the controls (37 %) (Table 5).

**Table 5:** Incremental distribution and overall LoS in patients with both low GSES (≤ 29) and high TSK (≥ 40)

Control group								
	LoS ≤ 5 days				LoS 6–9 days			LoS >10 days
GSES ≤ 29 and TSK ≥ 40	*n* = 24	9 (37 %)			10 (42 %)			5 (21 %)
Intervention group								
GSES ≤ 29 and TSK ≥ 40	*n* = 25	15 (60 %)			9 (36 %)			1 (4 %)
Overall LoS in the groups with low GSES ≤ 29 and high TSK ≥ 40								
		Mean LoS	SD	Range	Difference	95 % CI		*P*-value
Control Group	*n* = 24	7.63	4.24	18 days	2.15	0.24	4.05	0.028
gPCC Group	*n* = 25	5.48	2.08	12 days				

LoS: Length of stay; GSES: general self-efficacy scale; TSK: Tampa Scale of Kinesiophobia; gPCC: Gothenburg person-centred care; SD: standard deviation

The ASA grading system is used by anesthetists for grading patients’ physical health status (risk estimation) before surgery. In a simplified description the grades are ASA 1 = normal health, ASA 2 = mild systemic disease, ASA 3 = Severe systemic disease [33].

In the standardized care group the LoS was predicted for all patients to be 4–5 days and the patients graded ASA 1 would be most likely to achieve that, however 29 % did not manage that.

In the control group there was strong a significant correlation between age and ASA grade, age increased with risk (*p* = 0.01). A second strong correlation was found among the controls between ASA grade and LoS (*p* = 0.000): the trend was that LoS was increasing with impaired health. These trends were not observed in the intervention group, where a large reduction in LoS was seen among ASA grade 3 patients compared with ASA grade 1 and 2 patients (Table 6).

**Table 6:** Incremental distribution of LoS in patients by ASA grade (1–3)

Control Group *N* = *138*									
	LoS ≤ 5 days					LoS 6–9 days			LoS ≥ 10 days
ASA *grade* 1	*n* = 37	26 (70 %)				9 (24 %)			2 (5 %)
ASA *grade* 2	*n* = 83	45 (54 %)				27 (35 %)			11 (13 %)
ASA *grade* 3	*n* = 18	4 (22 %)				6 (33 %)			8 (44 %)
Intervention group *N* = *107* ^a^									
ASA *grade* 1	*n* = 21	14 (67 %)				7 (33 %)			0
ASA *grade* 2	*n* = 61	35 (57 %)				22 (36 %)			4 (7 %)
ASA *grade* 3	*n* = 25	18 (72 %)				2 (28 %)			0
Overall LoS for patients with ASA grade 1									
		Mean LoS	SD	Range	Diff	Age $\overset{\hbox{--} }{\mathrm{x}}$ (Median)	95 % CI		*P*-value
Control Group	*n* = 37	5.65	2.97	18 days	0.84	57 (58)	−0.58	2.26	0.28
gPCC Group	*n* = 21	4.81	1.72	9 days		67 (64)			
Overall LoS for patients with ASA grade 2									
		Mean LoS	SD	Range	Diff	Age $\overset{\hbox{--} }{\mathrm{x}}$ (Median)	95 % CI		*P*-value
Control Group	*n* = 83	6.65	3.81	25 days	0.81	67 (67)	−0.3	1.93	0.15
gPCC Group	*n* = 61	5.84	2.52	13 days		76 (78)			
Overall LoS for patients with ASA grade 3^b^									
		Mean LoS	SD	Range	Diff	Age $\overset{\hbox{--} }{\mathrm{x}}$ (Median)	95 % CI		*P*-value
Control Group	*n* = 18	11.50	9.36	40 days	6.78	73 (73)	2.94	10.62	0.001
gPCC Group	*n* = 25	4.72	1.57	7 days		69 (71)			

^a^There were 21 missing values in the intervention group because the ASA grade was not documented in these patients’ journals or on the anaesthesia form

An analysis of the missing patients’ characteristics did not show any significant differences compared with the other patients in the intervention group. Age range, 46–86 years; mean, 68 years LoS range, 2–8 days; mean, 4.3 days; SD, 1.6

^b^A correlation was found between GSES and LoS in the control group (*p* = 0,000)

LoS length of stay, ASA, American society of Anaesthesiologists’ classification system, gPCC Gothenburg person-centred care, SD standard deviation

## Discussion

The main finding in the present study was that low GSES and high TSK scores were common in patients scheduled for THA surgery, around 30 and 50 % respectively. The LoS for patients who had low GSES and high TSK scores was possible to affect. The hypothesis that a low self-efficacy and/or high fear of movement could disturb rehabilitation after surgery may be true. There is very little research connecting rehabilitation to GSES. In one study, patients’ perceived self-efficacy was found to have a predictive value regarding their return to acceptable levels of physical activity and muscle function 1 year after knee ligament reconstruction [34]. Another study showed that self-efficacy significantly increased during the rehabilitation process [35].

The level of pain after THA could be perceived as very high initially [18]. Based on previous knowledge low self-esteem and perception of pain is a signal to be still could have a negative impact on postoperative rehabilitation [32, 34, 36]. If the start of rehabilitation is delayed the LoS will be prolonged. In studies of Fast-track care systems patients who can start their rehabilitation already on the day of surgery, they can be discharged on day two or three [37].

As part of the preoperative investigation, an anaesthesiologist assessed and graded patients into different risk categories (1–3) to ensure that the surgery is medically safe. In clinical settings, it is not uncommon to regard patients in ASA category 3 (increased risk) as possessing less resources compared with patients graded as ASA category 1 or 2. In the present study, this was observed in the control group, where a correlation was found between LoS and ASA grade (*p* = 0.000). A larger reduction in LoS was seen with higher ASA grade in the intervention group, with the most dramatic reduction seen in ASA grade 3 patients. This was an important finding, indicating that it may be erroneous for health care professionals to assume that impaired health necessarily will interfere with patients’ abilities to achieve effective rehabilitation. In PCC there is a focus on the patients’ resources in order to detect possible deviations. A previous study evaluating patients with hip fractures found that, while the staff assumed that old age decreased patients’ abilities to achieve an effective rehabilitation, this was demonstrated to be incorrect [5]. In the gPCC intervention, patients were seen as partners and were expected to provide knowledge about themselves that was important for their care. This was meant to decrease the risk of ascribing impaired abilities they did not have.

In the control group an unexpected correlation was found between ASA grade 3 and low GSES. ASA grade is not commonly discussed with patients, which make this correlation difficult to explain. However, in an earlier study on patients with health impairments such as diabetes, heart disease or hypertension requiring joint replacements, patients felt that the health professionals marginalised their well-being and only focused on the joint surgery [38]. The results of the present study suggest that the person-centred intervention provided to patients did have a positive effect on tangible outcomes (LoS).

Adding tools such as GSES and TSK appeared to be useful and an important part of or complement to the PCC assessment. The instruments accentuated the heterogeneity among patients and helped discern and address the unique needs of each patient. The RN was able to develop a personal health plan together with each patient based on the patient’s narrative and the scores from the instruments. The health plan was a document meant to transfer information from the outpatient clinic to the hospital ward in order for RNs and physiotherapists to act upon. A previous study that evaluated patients who underwent THA found that shared decision-making and mutual respect did have positive effects on patient outcomes [39]. Higher quality of care, reduced postoperative pain, and improved physical function were also reported. The authors concluded that care focusing too much on flow and pathways cannot replace relational communication and the partnership between health care professionals and patients [39].

### Limitations

The study was conducted using a quasi-experimental prospective design in which consecutive patients in an intervention group were compared with patients in a control group receiving the usual care. This design was used primarily to avoid to the difficulty of having staff work with two care systems simultaneously. A disadvantage of this design is that it precludes evaluation of the true effects of an intervention; i.e., it is not possible to definitively conclude whether between-group differences occur because of the intervention itself or secondary to other unknown factors. However, since there are few circumstances under which non-RCT designs can yield reliable estimates of effect, conclusions from such studies, as ours, should be made with caution. Although random assignment was not applied, the control and intervention groups in this study were recruited according to the same protocol. In order to strengthen the study design a large number of clinical and socio-demographic variables were compared between groups.

In order to be informed of important decisions or changes that could have a favorable or unfavorable influence on the study, a study nurse participated in meetings with the surgeons and also in other important meetings. No such decisions occurred during the time for the study. During the intervention, recruitment stopped for several months because of the summer closedown and a shortage of nurses. However, these interruptions did not appear to cause bias in any of the measured variables.

## Conclusion

The GSES instrument and the TSK instrument were found useful as tools to detect vulnerable patients and the instruments enabled the development of individualised, person-centred rehabilitation plans. By giving patients, with low self-efficacy and/or high fear of motion, individual support and attention regarding the surgical procedure and the rehabilitation it seem to be possible to reduce their hospital length of stay. Finally, we found that patients with impaired health (i.e., ASA grade 3) had a significant correlation with low GSES. Probably these patients can gain the most from an intervention such as this.

### Relevance to clinical practice

In the present study, awareness of the patients’ who report low self-efficacy and high levels of fear of movement can help RNs tailor care more effectively in person-centred health plans. Our results suggests that short questionnaires like the GSES and TSK could be used routinely for planning the rehabilitation after THA surgery and likely for other surgical procedures as well, the ASA classification assigned by the anaesthetists is not a useful tool to predict patients’ resources for rehabilitation or hospital LoS. The study was quasi experimental and more studies are needed to confirm our results.

## Abbreviations

ASA classification: American society of anaesthetists’ classification
gPCC: Gothenburg person-centered care
GSES: General self-efficacy
LoS: Length of stay (in hospital)
RN: Registered nurse
THA: Total hip arthroplasty
TSK: Tampa scale of kinesiophobia
